# Characteristic and phylogenetic analyses of chloroplast genome for an endangered species *Manglietia lucida* (Magnoliaceae)

**DOI:** 10.1080/23802359.2019.1627927

**Published:** 2019-07-11

**Authors:** Yang Yang, Yan Zhao, Li-Ming Zhao, Shui-Lian He

**Affiliations:** a Yunnan Key Laboratory of Biomass Big Data, Yunnan Agricultural University, Kunming, China;; b College of Science, Yunnan Agricultural University, Kunming, China;; c College of Horticulture and Landscape, Yunnan Agricultural University, Kunming, China;; d Yunnan Academy of Forestry, Kunming, China

**Keywords:** *Manglietia lucida*, endangered species, chloroplast genome, phylogeny

## Abstract

*Manglietia lucida* is a threatened horticultural plant in Yunnan province of China. Now, the complete chloroplast (cp) genome of *M. lucida* was assembled. The cp genome of *M. lucida* was 160,134 bp in length and contained two short inverted repeat regions (26,595 bp), which were separated by a small single copy region (18,825 bp) and a large single copy region (88,119 bp). The cp genome encodes 108 unique genes, including 74 protein-coding genes, 30 transfer RNA genes and 4 ribosomal RNA genes. The topology of the phylogenetic tree showed that *M. lucida* was not formed a monophyletic clade but clustered together with genus *Magnolia*.

*Manglietia lucida* is an evergreen native tree of Magnoliaceae and native to Yunnan province in China. It has high ornamental value with large green leaves and showy elegant flowers (Chen and Yang 2010). Now, *M. lucida* is ranged as national first protection species and is becoming endangered in China. Hence, it is necessary to study the important species urgently. On the other hand, the origin of the taxa is not accurately resolved and taxonomical position is still unclear. Hence, a better understanding of this species has a great potential in species discrimination and protection of species.

In the present study, applying the Illumina technology, the whole chloroplast genome of *M. lucida* was sequenced, assembled, and annotated. The resultant data have been made publicly available as a resource for genetic information for *Manglietia* species and will provide a valuable plastid genomic resource for the future genetic and phylogenetic studies about *M. lucida.*

The fresh leaves of *M. lucida* were collected from the arboretum of Chinese Academy of Forestry (25.16° N, 102.75° E). The voucher specimen was deposited at Herbarium, Kunming Institute of Botany, CAS (KUN). Total genomic DNA was isolated from fresh leaves using a DNeasy Plant Mini Kit (QIAGEN, Valencia, California, USA) according to the manufacturer’s instructions for construction of chloroplast DNA libraries. The Illumina sequencing was conducted by Shanghai Genesky Biotechnologies Inc. (Shanghai, China). Resultant clean reads were assembled using GetOrganelle pipeline (https://github.com/Kinggerm/GetOrganelle). The genome was automatically annotated by using the CpGAVAS pipeline (Liu et al. 2012) and start/stop codons and intron/exon boundaries were adjusted in Geneious R11.0.2 (Biomatters Ltd., Auckland, New Zealand). All the contigs were checked against the reference genome of *Magnolia dandyi* (NC037004).

The complete chloroplast genome of *M. lucida* was 160,134 bp in length (Genbank accession number: MK764538). It was the typical quadripartite structure and contained two short inverted repeat (IRa and IRb) regions (26,595 bp) which were separated by a small single copy (SSC) region (18,825 bp) and a large single copy (LSC) region (88,119 bp). The chloroplast (cp) genome encodes 108 unique genes, including 73 protein-coding genes, 30 transfer RNA (tRNA) genes, and 4 ribosomal RNA (rRNA) genes. Twenty-one gene species are partially or completely duplicated, including seven PCG (*ndhB*, *rpl2*, *rpsl23*, *rps12*, *rps7*, *ycf1*, and *ycf2*), seven tRNA (*trnI-GAU*, *trnA-UGC*, *trnL-CAA*, *trnI-CAU*, *trnR-ACG*, *trnV-GAC*, and *trnN-GUU*) and all four rRNA (4.5S, 5S, 16S, and 23S rRNA). The overall GC content of the cp genome was 39.3%, while that of LSC, SSC, and IR regions was 38.0, 34.2, and43.2%, respectively.

The chloroplast genome sequences of Magnoliaceae were downloaded from GenBank and aligned with *M. lucida* using MAFFT (Katoh and Standley 2013) in Geneious R11.0.2 (Auckland, New Zealand). To resolve its phylogenetic placement within the family Magnoliaceae, the maximum-likelihood (ML) phylogeny tree was reconstructed using RAxML version 8.1.1179 (Stamatakis 2014). *Calycanthus floridus* var. *glaucus* (NC004993, Calycanthaceae) was selected as the outgroup. The topology of the phylogenetic tree showed that the species of *M. lucida* did not form a monophyletic clade but was clustered together with genus *Magnolia* (Figure 1). The complete cp genome information reported in this study will be a valuable resource for future studies of the species’ genetic diversity, and the conservation of this highly endangered species.

**Figure 1.**
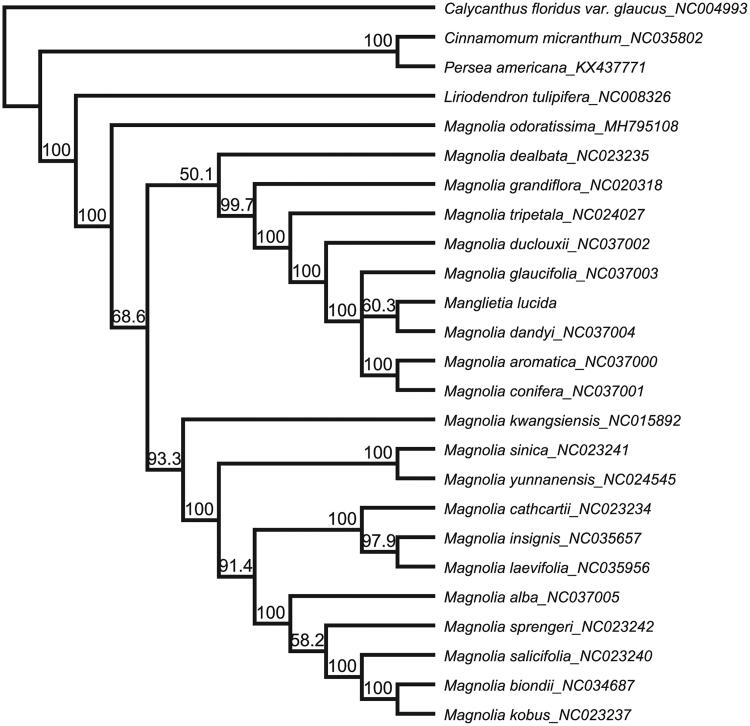
The maximum-likelihood (ML) phylogenetic tree based on 26 complete chloroplast genome sequence. Numbers at the right of nodes are bootstrap support values.
